# Health worker text messaging for blended learning, peer support, and mentoring in pediatric and adolescent HIV/AIDS care: a case study in Zimbabwe

**DOI:** 10.1186/s12960-019-0364-6

**Published:** 2019-06-07

**Authors:** V. Bertman, F. Petracca, B. Makunike-Chikwinya, A. Jonga, B. Dupwa, N. Jenami, A. Nartker, L. Wall, L. Reason, P. Kundhlande, A. Downer

**Affiliations:** 1 International Training and Education Center for Health (I-TECH), Department of Global Health, University of Washington, Seattle, WA USA; 2 Zimbabwe Ministry of Health and Child Care, Harare, Zimbabwe; 3 Department of Global Health E-learning Program, University of Washington, Seattle, WA USA

**Keywords:** E-learning, Text messaging, SMS, WhatsApp, HIV/AIDS, Blended learning, Healthcare worker training

## Abstract

**Background:**

In sub-Saharan Africa, shortages of trained healthcare workers and limited resources necessitate innovative and cost-effective approaches for training, supervising, and mentoring. This qualitative case study describes participants’ and trainers’ perspectives and experiences with a text messaging component of a blended training course in HIV counseling and testing in Zimbabwe, using minimal resources in terms of staff time and equipment requirements. This component included a whole-group discussion forum as well as two-person partner discussions designed to promote reflection and analysis, teamwork, and active learning.

**Case presentation:**

The Ministry of Health and Child Care (MoHCC) of Zimbabwe collaborated with the International Training and Education Center for Health (I-TECH) on adaptation of a 5-day in-service training in HIV Testing Services for Children and Adolescents. The new 7-week blended format included in-person sessions, tablet-based self-study, and discussions using the text messaging application, WhatsApp. Between August 2016 and January 2017, 11 cohorts (293 participants in total) were trained with this new curriculum, incorporating text messaging to support peer-to-peer and work-based education.

Data collected included training participants’ feedback, key informant interviews with the training team, and thematic analysis of WhatsApp messages from full-cohort discussions and a sampling of one-to-one partner discussions.

A total of 293 healthcare workers from 233 health facilities across all provinces in Zimbabwe completed the blended learning course. Participants strongly endorsed using WhatsApp groups as part of the training. In the whole-group discussions, the combined cohorts generated over 6300 text messages. Several categories of communication emerged in analysis of group discussions: (1) participants’ case experiences and questions; (2) feedback and recommendations for work issues raised; (3) inquiries, comments, and responses about course assignments and specific course content; (4) encouragement; and (5) technical challenges encountered using the blended learning methodology. Case discussions were complex, including patient history, symptoms, medications, and psychosocial issues—child abuse, adherence, and disclosure.

**Conclusions:**

Using text messaging in a communication platform that is an ongoing part of healthcare workers’ daily lives can be an effective adjunct to in-service training, minimizing isolation and providing interactivity, supporting students’ ability to fully integrate content into new skill attainment.

## Introduction

A critical shortage of trained health workers impacts the ability of many countries in sub-Saharan Africa to reach UNAIDS global HIV/AIDS targets by 2020—to diagnose 90% of all HIV-positive persons, provide antiretroviral therapy (ART) for 90% of those diagnosed, and achieve viral suppression for 90% of those treated (the “90-90-90” targets). The burden of HIV and health worker shortage has led to the practice of task shifting—delegation of medical and health service responsibilities from higher to lower cadres of health staff, in some cases non-professionals [1]. To address the HIV epidemic in Zimbabwe, the Ministry of Health and Child Care (MoHCC) has undertaken strategies that include prevention, treatment, care, and support services.

In countries with limited resources, including Zimbabwe, addressing health worker shortages and service needs necessitate innovative, cost-effective approaches for training, supervising, and mentoring healthcare workers. These approaches need to minimize the burden on an already-overwhelmed healthcare system by limiting the time health providers spend away from work posts to attend training, while still providing the instructional experiences that trainees need to perform their jobs well [2]. This report describes one component of a blended instructional approach, using text messaging to support a self-study training for counselors in HIV counseling and testing.

Blended learning combines a variety of learning methods (such as in-person, online, print, and social media) and learning environments (such as instructor-led, teamwork, peer-to-peer, self-study, and individual work) [3, 4]. Growing evidence supports the effectiveness of the blended learning approach for health worker training and suggests that it is well received and may be a more affordable instructional method when compared with standard classroom instruction [5–8].

Data suggest that students benefit from an environment where a mixture of classroom and online technologies is employed, promoting ongoing interest in the subject matter. Applied and interactive learning have been found to be critical for effective clinical training, so it is important to assure that these aspects of training are not lost in the development of blended learning courses. As Joynes [3] has stated in a systematic review of distance and blended learning training for healthcare workers, “...methods that actively involved learners interacting with learners in a clinical environment were theoretically and empirically superior in their educational effectiveness as compared to either classroom-based activities and/or didactic activities….”

Peer-to-peer interaction through email or online discussion forums increases opportunities for reflection and analysis, supports teamwork, and moves learners from passive to active learning [9, 10]. Training programs that include significant components of work-based training support learners in attainment of competencies and the application of behavior change due to deeper understanding of content and the opportunity for students to reflect on their own learning and perceptions [3].

One low-cost approach to incorporating interaction into blended learning is through text messaging (also known as “short message service” or SMS). Literature is minimal regarding the use of SMS as an interactive component of blended learning in healthcare worker training, although this modality is increasingly being employed in various contexts, including university coursework [11], health worker supervision [12], military physician mentoring [13], and direct patient support [14]. Suggested benefits of incorporating social media into healthcare worker training include workers’ familiarity with this technology, ready access to it, and frequency of use [15], user satisfaction [16], and improved knowledge and confidence [17].

## Background

In Zimbabwe, primary counselors (PCs) are a cadre of health worker that has been established to support task shifting, providing HIV testing and counseling (HTS) to adult, adolescent, and pediatric patients. This cadre of health worker is secondary school educated, a minimum of 25 years of age, and, in general, has no formal medical education prior to being employed as PCs. These health workers are the target audience for a blended learning course in HTS for children and adolescents.

Healthcare worker training in HIV counseling skills for children and adolescents has been identified as a critical component of strengthening efforts towards reaching the 90-90-90 targets [18], as HTS is an entry point to HIV prevention, treatment, care, and support. Zimbabwe’s national adult prevalence of HIV is estimated at 14%, with an estimated 33 000 new infections annually and a mother-to-child transmission (MTCT) rate of 5.2%. For children and adolescents, the prevalence rate is estimated at 0.9% for infants aged 0 to 17 months, 0.8% for those 18 to 59 months, 1.7% for children 5 to 9 years old, 2.5% for children 10 to 14 years old, and 3.6% for adolescents 15 to 19 years of age. Viral suppression is estimated at approximately 50% for children 5 to 19 years of age [19].

Counseling, care, and support are required by three main groups of children: children who have been confirmed HIV negative, children who have tested HIV negative yet remain HIV exposed or at risk of HIV transmission, and children who have been confirmed HIV positive. Children and adolescents present unique challenges that differ from those of adults, further underlying the need for healthcare worker training which specifically targets the counseling and testing of young people. Common counseling issues for children and adolescents may include developmentally diverse physical and psychosocial needs and communication abilities, family challenges and housing instability, abuse, disclosure, legal and ethical issues surrounding age of consent and the principle of best interest of the child; the need to equip children and their caregivers with accurate information about their condition and the implications of living with HIV; and coping with stigma and discrimination [20, 21].

## Case presentation

This case study describes a training intervention using WhatsApp, a mobile phone text messaging application that is in widespread use worldwide [22], as one component of a blended learning program. The course was designed to build the skills and knowledge of PCs in HIV-related counseling for children and adolescents. Questions being addressed include the following: Did trainees participate in the text messaging groups and in what ways? What were the perceptions of trainees and trainers on the text messaging component of the training? What lessons could be learned from their experiences to apply to future use of this instructional method?

Zimbabwe’s MoHCC developed the HTS for Children and Adolescents guidelines and accompanying in-service training to address the needs and challenges related to identifying HIV-infected youth [23]. In collaboration with MoHCC, The International Training and Education Center for Health (I-TECH) adapted the 5-day in-service training to a 7-week blended training that included tablet-based self-study, a one-and-a-half-day classroom session at the beginning of the course, and a 4-hour classroom session at the end. The self-study component included videos, podcasts, case studies, quizzes, and self-reflection questions. These activities were complemented by the SMS component, including a whole-group discussion forum as well as two-person partner discussions. The course was facilitated by three trainers who presented content, managed logistics, and interacted with the WhatsApp groups. Staffing did not include an information technology manager; participants were invited to send messages or call one of the trainers if technical issues arose.

Participants were expected to communicate with their partner, using their personal mobile devices and SIM cards, via WhatsApp each week, and the entire class cohort participated in the group WhatsApp discussion. At the end of each week, the pairs were required to forward their discussions to the course facilitator, who awarded course credit based on timeliness, relevance of the case to the week’s topic, and responses received from the partner. The course facilitators used WhatsApp to make announcements and send encouragement and reminders about the weekly assignments and to provide feedback and suggestions in response to participants’ cases. Participants who completed all assignments and attended the face-to-face sessions were given certificates at the end of the training.

Between August 2016 and January 2017, a total of 11 cohorts were trained with this new curriculum, incorporating WhatsApp to support peer-to-peer and work-based education.

## Methodology

This SMS intervention was one component of a formative assessment of blended learning in Zimbabwe, which aimed to gather information on target audiences, needs, barriers, and facilitators to effectively implementing healthcare worker training using this new and innovative approach. The formative assessment was conducted in collaboration with the Zimbabwe MoHCC and was approved, with a non-research determination, by institutional review boards at the University of Washington, CDC Zimbabwe, CDC Atlanta, and the Medical Research Council of Zimbabwe.

To evaluate the SMS component of the blended learning program, qualitative data were collected from 10 of the 11 cohorts; one group’s data was incomplete due to inadequate memory on the device. The data included:

- Participants’ feedback provided during debrief discussions with peers (*n* = 293), trainers (*n* = 2), and administrators (*n* = 1), at the final face-to-face classroom sessions between October 2016 and January 2017;
- Key informant interviews conducted in June 2017 with the training team (*n* = 3) who ran the program; and
- Thematic analysis of WhatsApp messages, from full-cohort discussions and a sampling of one-on-one partner discussions.

For all data, no individual identifiers were stored with data to ensure confidentiality of participant information. Verbal consent was obtained from respondents to participate in the study. Key informant interviews were conducted with program trainers. Interviews were recorded and notes were taken. For debrief discussions and interviews, facilitators followed a semi-structured format and used a discussion guide that included questions about participants’ experiences with the training modalities, as well as about the training content—what went well, what they learned, and challenges they experienced. Each debrief session was conducted by a facilitator and an additional evaluator who took notes on participant responses.

For analysis of the full-cohort and partner discussions, the text messages were extracted from the administrator’s device, uploaded in a Microsoft Word file to a password-protected folder, and analyzed using Atlas.ti software. Thematic analysis of all data was conducted by two members of the evaluation team who were blind to the identities of the participants, and analysis followed a general inductive approach [24]. Starting with the primary focus of the evaluation—identifying needs, barriers, and facilitators to using WhatsApp in this context—a first reviewer read all materials and developed a list of themes identified in the material. Next, each theme was reviewed, and sub-themes were identified, with reviewers discussing and reaching consensus on the assignment of thematic codes to the text. Representative quotes were selected for each theme and sub-theme.

The two different types of discussions—group discussions and partner discussions—were coded and analyzed separately. All 10 cohorts’ group discussions were analyzed, in addition to a random sample of 20 of the partner discussions.

## Results

A total of 293 healthcare workers from 233 health facilities across all provinces in Zimbabwe completed the blended learning course. Ninety percent of the participants were primary counselors, and 10% were nurses who serve as supervisors to primary counselors. Sixty-three percent of participants were female.

Analysis focused on the acceptability, needs, barriers, and facilitators of using SMS as an adjunct to the blended training course. Broad themes related to this focus that emerged in the analysis included positive feedback on the acceptability and use of text messaging as an adjunct to the training course, and extensive use of the platforms for information seeking, support, and case discussion.

All quotes presented in this report were not edited for grammar, spelling, or completeness; however, some were edited for brevity and, where text was extracted, it was replaced with ellipses (“…”).

### Acceptability and use of text messaging as an adjunct to the training course

Facilitators’ post-course debriefing feedback indicated that participants strongly endorsed using WhatsApp groups as part of the training. As one trainer described it, “*Most were thrilled*.” In addition to general feedback that it was helpful, positive comments included perceptions that the WhatsApp discussion groups helped participants identify their own information gaps; that comments about cases shared by other participants provided learning opportunities for everyone; and that the discussion groups provided access to supervisors, trainers and individuals with greater experience.

Feedback from training administrators similarly indicated that the text messaging component was well received by training participants. They reported a perception that participants’ messages showed skill improvement over time. For example, one reported, “*Knowledge gap in the appropriate application of skills and techniques improved as the course progressed.*” Comments extracted from the text messaging discussion transcripts echoed these sentiments: “*We are learning really, today I was so excited on the way I handled a counselling session of a child … guys you feel good when you know what you are doing thanks BL HTS Pilot group.*” And, “*Thanks a lot to everyone in this group I’ve learnt a lot from your discussions.*”

Course participants were actively involved in both the partner discussions, which were required, and whole-group discussions, which were optional. In the whole-group discussions, the combined 10 cohorts generated over 6300 text messaging entries; many of these discussions continued after the 7-week course was concluded, and all tablets were returned undamaged. Ongoing post-course communications included social interactions and check-ins, as well as users’ real-time usage of the platform to discuss workplace issues, cases, questions, advice, and support.

### Communication categories within group discussions

The group discussions were analyzed, and several broad categories of communication emerged. For this evaluation, the following categories were the primary focus of analysis: (1) participants’ own case experiences and questions about them; (2) feedback and recommendations for work issues raised; (3) inquiries, comments, and responses about course assignments and specific course content; (4) encouragement; and (5) challenges encountered and recommendations for using the blended learning methodology. In addition, the platform was used for greetings and social contacts. Examples from these categories are presented below.

### Participant’s workplace cases, feedback, and recommendations

Participants were required to use the text messaging partner discussions to present challenging work cases relevant to the module topics. Group discussions were open forums where participants had the option of presenting additional workplace challenges, requesting feedback, and asking questions as they desired. Analysis of the data from both types of discussion revealed that participants were able to use WhatsApp to describe cases’ complexity, with detail, including aspects of patient history, symptoms, medications, and psychosocial issues—such as child abuse, adherence, disclosure, and support systems—and legal and ethical matters.

*Participant 1: A girl 16 came at my clinic …[her grandma] accuses her of having of sleeping with boys ...both parants died she went to her stepmother who came with her at the clinic .she was crying .they came to the counselling room.i gave her tissues and offered thm seats … she stopped crying .and said she want to be tested to prove her grandma wrong*

*we discussed about hiv .results outcome .she consented to be tested .i tested her and her result were negative …*

*Participant 2: You need to discuss about SRH [sexual and reproductive health] issues its important she is sexual active*

*Participant 3: How about window period,Social welfare Childline,SRH,widen system,dig for more information.Not bad.*

*Participant 4: Also give her the infomation that she is at risk though her HIV results are negative*

*Participant 1: Thank you guys for your response*

*Participant 5: Is she sexually active or they are just suspicious? If sexually active there is need for counseling on SRH issues as stated already*

*Needs to know dangers of unprotected sex, discuss about family planning methods, screening for STIs, screening for cervical cancer...*

*Participant 6: I think you need to assess the girl’s support system through widening the system nd explore ...then you also need to assess what the girl feels nd then discuss about SRH issues taking note that every individual have unique feelings.*

### Inquiries, comments, and responses about course assignments and specific course content

Participants used the group discussion forum to ask questions about course content, as in the example below. Often, several participants responded, some corrected others’ incorrect responses, and, at times, a facilitator provided correct information when there was confusion.

*Participant 1: Guys help me what is perinatal*

*Participant 2: Death of the infant soon after delivery with 72 hours in Maternity.*

*Participant 3: Perinatal is not death.its a period immediately before and after birth*

*Participant 3: Usually between 20-28 weeks of pregnancy to 4_6weeks after birth*

*Participant 3: Death of an infant within 72hrs is early neonatal death*

### Encouragement

Facilitators offered motivation to participants in addition to using the WhatsApp discussion groups to deliver messages about the course and its requirements. For example:

*Facilitator: Thank you for participating in our tablet self study programme. We hope that you will have a good experience with this training and that you will gain new knowledge and skills to use when working with children, families and adolescents. Empowering children and adolescents to take part in their own health care will help play a role in their retention in care. As Archbishop Desmond Tutu said, “Give young people a greater voice. They are the future and they are much wiser than we give them credit for.”*

Facilitators also provided encouragement and positive feedback in response to participant posts:

*Facilitator:*  *for the job well done. Especially use of soldier game. Well done! With your support he will heal.*

Participants gave encouragement to each other. In this example, participants provided suggestions while offering positive feedback and encouragement:

*Respondent 1: Sounds like a good session to me, well handled. However I think you should have referred to a support group if there are any around or at least try to widen his support system among his peers.*

*Respondent 2: I acknowledge involving his Headmaster but mind you he is a youth and identifies best with his peers, a friendlier approach like raising awareness might do more good than punishments and disciplining his peers. I hope this helps your case. Good day.*

*Respondent 2: I should applaud you for taking the first plunge into the Group discussion. Thank you.*

*Respondent 3: Job well done, and most importantly refer him to an active support group that wil make him cope and understand he s not the only adolescene on ART [antiretroviral therapy].*

*Respondent 4: Well done … you did very well for motivation reassuarring to discuss about disclosure to the school authorities.*

### Non-work-related content

In addition to the primary communication categories reviewed, it was also observed that the group forum was used for posting an assortment of non-work-related content, including jokes, stories, ads, religious messages, or articles. Many of them included emoticons as seen in the example that follows:

... *LET'S MAKE IT A HABIT*   * Have you ever wondered what would happen if we treated the bible the way we treat our*   *mobile phone...? What if we can carry it with us wherever we go; In our bags*   *.... & our pockets?...*

### Challenges using SMS as a part of blended learning

Participant debrief sessions as well as analysis of the message content yielded information about the challenges encountered employing this modality. These included resource issues, including power cuts which made it difficult to recharge the tablets, resulting in delays in adhering to assignment deadlines. In many regions of the country, there is inconsistent mobile phone reception related to the stability of the telecommunications companies. In addition, some participants reported that their phones had limited space for typing case descriptions.

Other challenges reported during the debrief sessions included difficulty balancing work and personal responsibilities with the time needed to complete the course. Some individuals reported frustration that their WhatsApp discussion partners were slow to respond and that they had to turn in their material to the course administrator before the discussion was complete. Technical challenges observed from analysis of the group and partner discussions included a lack of “threading” of case discussions. It was observed that some of the group conversations were broken up, with multiple cases overlapping. At times, this issue resulted in difficulty following a conversation.

## Discussion

The primary questions for this study were the following: Did trainees participate in the text messaging groups and in what ways? What were the perceptions of trainees and trainers on the text messaging component of the training? What lessons could be learned from their experiences to apply to future use of this instructional method? Results suggested that trainees participated actively in the partner and group discussions, describing complex cases, eliciting input from fellow participants, and providing support to each other. The feedback received from trainees and trainers suggested a strong endorsement of this methodology. Analysis of the message content showed that the breadth and complexity of the topics discussed was accommodated using SMS group and partner discussions; participants openly shared and discussed very sensitive case information, while protecting client anonymity. This suggests that participants were comfortable using this platform for peer support, learning, and problem-solving.

The favorable response and wide adoption of use of SMS seen in this case are consistent with findings of a consensus group on in-service training for healthcare workers, in which key priorities for successful instruction included using “accessible learning platforms and mechanisms that can be efficiently updated in a timely manner, in addition to print-based materials.” The recommendations of the consensus group also included prioritizing “time efficiency in the design, planning and mode of delivery of training to prevent or minimize health worker absenteeism.” Plans for this course incorporated these themes, acknowledging that WhatsApp was a widely used and accessible platform among this cadre [25].

Although research to date is limited on the use of WhatsApp to support training for healthcare workers, some studies have begun to explore this modality. Employing WhatsApp, Goyal et al. [16] found a majority of participants in a pathology residents’ WhatsApp discussion in Delhi, India, reported the discussion group was useful. In addition to user satisfaction, some studies indicate positive practice outcomes. Bakshi and Bhawalkar [17] reported positive outcomes of a 3-month WhatsApp discussion group among Korean anesthesia residents, indicating that participants improved in knowledge, confidence, and documentation practices.

The positive results seen in this case study indicate that self-study training using tablets, combined with SMS discussion, may be a useful blended learning approach. Incorporating SMS into the training for partner activities and discussion groups can provide the individual learner with opportunities to minimize isolation [26], provide interactivity, and integrate content into new skill attainment. The SMS component also provides the entire group with peer interaction to enhance and build on the real-life situations they face at work. It is a low-cost platform—one that is accessible, readily used, and has the potential to support the building of healthcare worker knowledge and skills in low-resource settings.

## Limitations

This was a program evaluation, embedded within an ongoing and evolving program overseen by Zimbabwe’s MoHCC. Limitations include the potential for positive response bias: Respondents may have sought to please trainers and evaluators asking for feedback, providing more positive responses than they might otherwise. To minimize this, respondents were reminded of the formative nature of the project and questions and were encouraged to respond honestly. The scope and qualitative case study design of the present study limit generalization to other contexts, as this project focused on one course and one health worker cadre within the context of Zimbabwe’s HIV program. This was not an impact study on the effectiveness of the training overall or of the SMS component’s contribution to training outcomes; therefore, conclusions drawn from this study are limited to questions regarding use and acceptability of this method.

## Conclusions

The positive results seen in this case study indicate that SMS group and partner discussions were well received and may be valuable components of a blended learning program. In the context of ongoing resource limitations for in-service training, as is seen in Zimbabwe, it is important to support learning experiences for healthcare workers who have complex responsibilities, such as those of the primary counselors participating in this course. Further research may provide additional information about best practices for using SMS as an effective instructional modality in varied settings and for varied training topics, and outcome studies are warranted to yield further evidence to guide program design.

## Abbreviations

ART: Antiretroviral therapy
HTS: HIV testing and counseling
I-TECH: International Training and Education Center for Health
MoHCC: Ministry of Health and Child Care
MTCT: Mother-to-child transmission
PC: Primary counselor
SMS: Short message service
